# Efavirenz-Based Highly Active Antiretroviral Therapy Disrupts Folliculogenesis: Evidence From 48 Women of Reproductive Age

**DOI:** 10.7759/cureus.79597

**Published:** 2025-02-24

**Authors:** Aigbe G Ohihoin, Henry E Ogbeifun, Esther Ohihoin, Airat Bakare, Imuwahen Mbarie, Ebiere Herbertson, David Oladele, Olufemi M Omololu, Osagie Cole, Vahe Ohihoin, Agatha Wapmuk, Onwuamah Chika, Oliver Ezechi, Babatunde L Salako, Simon D Taylor-Robinson

**Affiliations:** 1 Department of Cell Biology and Neuroanatomy, Mellowes Center for Genomic Sciences and Precision Medicine, Medical College of Wisconsin, Sussex, USA; 2 Department of Clinical Sciences, Nigerian Institute of Medical Research, Lagos, NGA; 3 Department of Physiology, Eko University of Medicine, Lagos, NGA; 4 Research and Development Unit, HICI Healthcare Limited, Lagos, NGA; 5 Graduate School of Biomedical Sciences, University of Texas at Austin, San Antonio, USA; 6 Research and Development Unit, HICI HealthCare Limited, Lagos, NGA; 7 Department of Anatomy, College of Medicine, University of Lagos, Lagos, NGA; 8 Institute of Child Health, University of Benin, Benin, NGA; 9 Department of Obstetrics and Gynecology, Lagos Island Maternity Hospital, Lagos, NGA; 10 Medical Service Branch, Headquarters Nigeria Airforce Abuja, Lagos, NGA; 11 Department of Microbiology, Nigerian Institute of Medical Research, Lagos, NGA; 12 Department of Surgery and Cancer, Imperial College London, London, GBR; 13 Department of Internal Medicine, Busitema University, Mbale, UGA

**Keywords:** anti-retroviral drugs, folliculogenesis, hiv, reproductive age, reproductive outcome

## Abstract

Introduction: Interventions to eliminate mother-to-child transmission of HIV have led to a low vertical transmission rate and improved reproductive outcomes for women living with HIV. There are genuine concerns about the long-term effect of antiretroviral drugs (ARVs) on the future reproductive function of females, as female rodent models have shown impaired folliculogenesis when treated with contemporary ARVs. There is a need to investigate the effect of contemporary ARVs on the ovarian reserve of patients receiving ARVs.

Methods: This cross-sectional study was conducted on women between the reproductive age of 15 and 45 years, forming three study categories. Group 1 comprised HIV-positive patients already on efavirenz-based antiretroviral therapy. Group 2 comprised HIV-positive patients who were naïve to ARVs at the time of recruitment. Group 3 comprised HIV-negative women within the reproductive age range not on ARVs (controls). We used t-test and ANOVA for statistical analysis. The alpha level was significant if the p-value was <0.05.

Results: The average value of follicle-stimulating hormone was significantly higher in the HIV-positive group receiving ARVs, compared to the control group (p = 0.039). The average value of luteinizing hormone was significantly higher in the HIV-positive group receiving ARVs when compared to the HIV-negative group (p = 0.014). The antral follicular count was significantly reduced in the group receiving ARVs, compared to HIV-positive individuals naïve to ARVs and HIV-negative controls (p = 0.009).

Conclusion: Evidence from this study suggests that efavirenz-based antiretroviral medication disrupts follicular development.

## Introduction

The use of highly active antiretroviral therapy (HAART) has led to improved outcomes for patients living with HIV/AIDS. As a result, HIV is now regarded as a chronic medical condition, rather than one with impending mortality. HAART regimens are antiretroviral drugs (ARVs) that consist of at least three different components with at least two of them having a different mechanism of action. There is evidence to suggest that the life expectancy of patients who are well-managed for HIV infection is not different from their HIV-negative counterparts [1,2]. Interventions to eliminate mother-to-child transmission of HIV have led to a very low vertical transmission rate and improved reproductive outcomes for women living with HIV [3,4].

There are, however, challenges with the long-term use of the anti-retroviral drugs, as side effects of these drugs have become issues of concern. For example, nucleoside reverse transcriptase inhibitors have been implicated in mitochondrial damage and hematological toxicity, leading to anemia [5-7].

Adverse effects related to the central nervous system have been implicated in non-nucleoside reverse transcriptase inhibitors [8,9]. There is evidence to suggest that some of the side effects can affect the reproductive efficiency of the patients as evidence abound that some ARVs can affect sperm motility in some males [10]. There is also the suggestion that ARVs can lead to testicular atrophy [11]. Animal models using female Wistar rats have shown impaired estrous cycle and impaired folliculogenesis in the animals treated with efavirenz-based HAART [12,13]. There is therefore the need to investigate the effect of contemporary ARVs on the ovarian reserve of patients receiving these agents. This study aimed to determine the antral follicular count and reproductive hormone profile of HIV-negative and HIV-positive women within the reproductive age group and ascertain the impact of HAART (ARVs) on folliculogenesis in HIV-positive women receiving these medications.

A major aspect of this manuscript was posted as a preprint in Research Square [14].

## Materials and methods

The study was conducted at the Nigerian Institute of Medical Research (NIMR) located in Yaba, Lagos, Nigeria, the Lagos Island Maternity Hospital (LIMH) located in Lagos Island, Lagos State, Nigeria, and HICI Healthcare Hospital and Diagnostic Clinic located in Lekki, Lagos, Nigeria. NIMR has a dedicated clinic for patients living with HIV, incorporating prevention of mother-to-child transmission (PMTCT) services and gynecological care for people living with HIV. LIMH is a dedicated tertiary reproductive health hospital offering obstetric and gynecological care to women. HICI Healthcare is a secondary-level private health facility known for ultrasound diagnostic services and echocardiography. Prior to the commencement of the study, ethics approval was obtained from the Institutional Review Board (IRB) of the NIMR (IRB/17/027). This approval covers all three participating institutions.

A cross-sectional study involving women within the reproductive age range of 15-45 years was conducted in these three centers between the period of September 2017 to December 2017. Data from these patients were collected prospectively after they met the inclusion criteria. The HIV-positive participants in the study were recruited from NIMR, while the HIV-negative participants were recruited from LIMH. Transvaginal ultrasonography was performed at the HICI Healthcare Diagnostic Center. The study population had three groups. Group 1 were HIV-positive females within the reproductive age range receiving HAART for their condition, group 2 were HIV-positive women with a new diagnosis of HIV not yet commenced on HAART, and group 3 were HIV-negative patients within the reproductive age range (the control group).

Cohort grouping

Inclusion Criteria for Group 1

Sexually active HIV-positive females within the reproductive age of 15-45 years who were on the HAART combination of lamivudine, tenofovir, and efavirenz for at least six months.

Exclusion Criteria for Group 1

The exclusion criteria for group 1 were HIV-positive women outside the reproductive age range, HIV-positive women who had not commenced HAART, HIV-positive women who were pregnant, HIV-positive women on fertility treatment, and women within the reproductive age range who were being evaluated for infertility.

Inclusion Criteria for Group 2

Sexually active HIV-positive women within the reproductive age range of 15-45 years with a new diagnosis of HIV and who had not been commenced on ARVs.

Exclusion Criteria for Group 2

The exclusion criteria for group 2 were HIV-positive women who were outside the reproductive age range, HIV-positive women who had been commenced on ARVs, HIV-positive women who were pregnant, HIV-positive women on fertility treatment, and women within the reproductive age range who were being evaluated for infertility.

Inclusion Criteria for Group 3

Sexually active HIV-negative women within the reproductive age range of 15-45 years and who were not being evaluated for infertility.

Exclusion Criteria for Group 3

The exclusion criteria for group 3 had HIV-negative women within the reproductive age range who were not sexually active and HIV-negative women within the reproductive age range who were being evaluated for infertility.

Sample size calculation

The sample size was determined using the following formula:

 $N = {Z^2 P q}/{D^2}$

Where, N = sample size; Z = Z value at 95% (0.95) confidence limit, read from a standardized normal distribution table; P = estimated prevalence based on previous studies; q = 1-P; D = precision; ​​​​​P = (prevalence of HIV in Lagos) = 1.4%; D = 50% (0.05); q = 0.98.

$N = 1.962 \times 0.02 \times 0.99 / (0.05)^2 = 16$ 

A total of 48 participants were therefore recruited for the three study groups.

Interventional agents

HAART, also known as antiretroviral drugs (ARVs), for this study was administered to the patients who satisfy the criteria as stated in the cohort grouping above. Efavirenz is a non-nucleoside reverse transcriptase inhibitor (NNRTI) used for the management of HIV/AIDS in our treatment clinic. This drug is used in combination with other drugs in a HAART (ARVs) combination. Efavirenz, tenofovir, and lamivudine were therefore administered in a single-dose combination for the conduct of this research work. A fixed-dose combination of efavirenz, tenofovir, and lamivudine manufactured by Mylan Pharmaceuticals Pvt Ltd (Mumbai, India) was the standard ARV therapy used. Tenofovir is a nucleotide reverse transcriptase inhibitor (NRTI) also used for the treatment of HIV/AIDS in a standard HAART combination with lamivudine (3TC), a nucleoside reverse transcriptase inhibitor.

Data collection

The nature of the research work was explained to all potential participants who satisfied the inclusion criteria, and all provided written consent. Anonymized socio-demographic data and clinical data were extracted through a proforma. Patients who registered at the NIMR clinic routinely had such anonymized data pooled into an electronic data bank, which was kept behind an electronic firewall in accordance with strict General Data Protection Regulation (GDPR) regulations. In this study, these sociodemographic and clinical data were obtained from the patients prospectively during clinical evaluation, after written informed consent had been obtained. After clinical evaluation, laboratory parameters such as CD4 count, HIV viral load, full blood count, serum electrolytes, urea, and creatinine were also obtained, with additional blood samples obtained for the estimation of the following hormones: follicle-stimulating hormone (FSH), luteinizing hormone (LH), and estradiol. All the hormones were measured using solid-phase enzyme immunoassays.

Ultrasound procedure for antral follicular count

The antral follicular count was performed during the early follicular phase of the menstrual cycle prior to ovulation. The preferred method for the determination of the antral follicular count was via a vaginal ultrasound scan at a frequency of 6-8 MHz. A Mindray M7 ultrasound machine (Mindray Inc., Shenzhen, China) was used for this procedure. The frequency of the vaginal transducer used for the procedure was 7 MHz. Patients were counseled for the procedure and additional consent was obtained further to the signed informed consent used at study recruitment.

Statistical analysis

The dataset had continuous variables, and these were reported as means and standard deviations. Analysis of variance (ANOVA) was used to compare continuous variables between groups since three groups were involved in the study. Statistical significance was set at a p-value less than 0.05.

Ethical consideration

Ethical approval was obtained for the conduct of the study from the Institutional Review Board (IRB) of the Nigerian Institute of Medical Research (NIMR) (IRB/17/027). The study complied with the precepts set out in the 1975 Declaration of Helsinki on Human Rights. Written informed consent was obtained from the patients.

## Results

The average age of the HIV-negative patients was 32 years (range: 23-42 years), while that of the HIV-positive patients not receiving HAART (ARVs) was 33.4 years (range: 18-45 years) and that of the HIV-positive patients in receipt of HAART (ARVs) was 37.58 years (range: 32-44 years). Across the study groups, none of the participants had been involved in smoking or significant consumption of alcohol. The average CD4 count was 725 cells/mm3 (range: 400-956 cells/mm3) and the average viral load was less than 20 copies/mL. The average duration of ARV use was 39 months.

**Figure 1:**
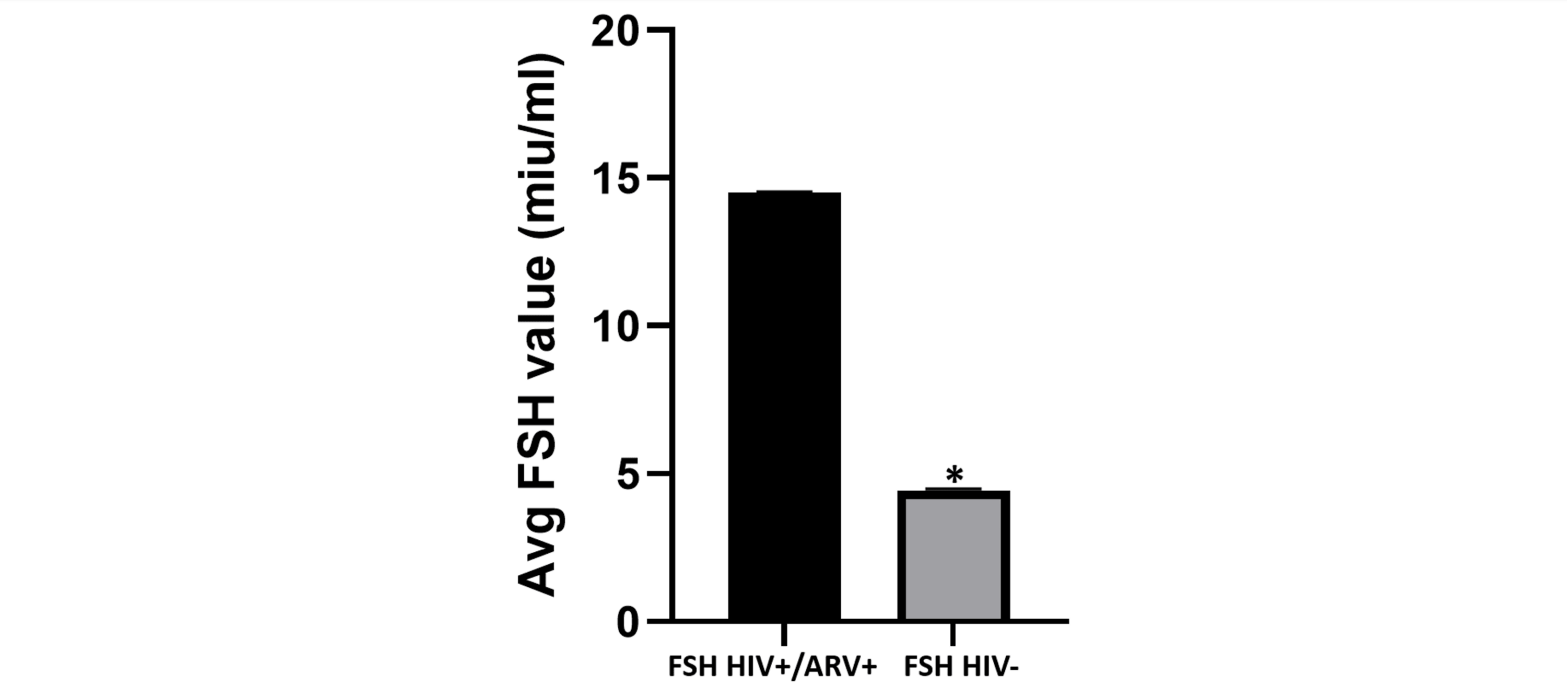
Average FSH value: HIV-positive participants on ARV vs. HIV-negative participants. The bar chart shows that the average value of FSH in HIV-positive patients on ARV is significantly higher than the average FSH value of HIV-negative patients. This difference is found to be statistically significant (p = 0.039). FSH: follicle-stimulating hormone; ARV: antiretroviral drugs.

**Figure 2:**
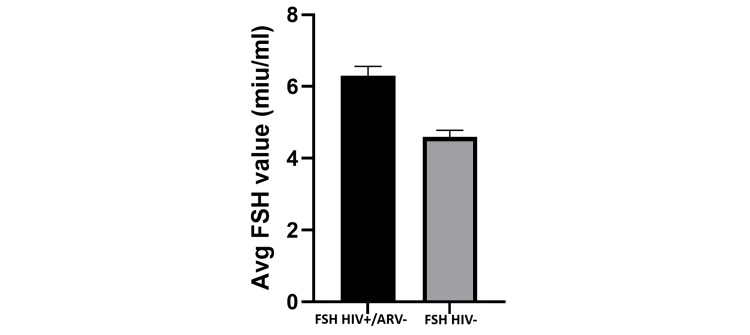
Average FSH value: HIV-positive participants not on ARV vs. HIV-negative participants. The bar chart shows that the average FSH value of HIV-positive patients not on ARV is higher than the average FSH value of HIV-negative patients; however, this difference is not statistically significant (p = 0.122). FSH: follicle-stimulating hormone; ARV: antiretroviral drugs.

In Figure 1, the average FSH value of HIV-positive patients on ARV was 14.80 miu/mL (range: 5.59-44.21miu/mL), while the average FSH value of HIV-negative individuals was 4.85 miu/mL (range: 1.76-7.92miu/mL). This difference was found to be statistically significant (p = 0.039). In Figure 2, the average FSH value of HIV-positive patients not on ARV was 6.10 miu/mL (range: 3.65-18.76 miu/mL), although this value was higher than the average FSH value of HIV-negative patients at 4.85 miu/mL. This difference was, however, not statistically significant.

**Figure 3:**
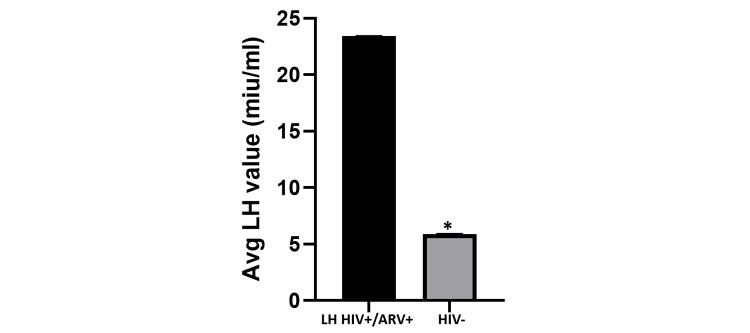
HIV-positive participants on ARV vs. HIV-negative participants. The bar chart shows that the average LH levels in HIV-positive patients on ARV are significantly higher than the average LH levels in HIV-negative patients. This difference is statistically significant (p = 0.014). ARV: antiretroviral drugs; LH: luteinizing hormone.

In Figure 3, the average LH value of HIV-positive patients on ARV was 23.85 miu/mL (range: 5.15-67.34 miu/mL), while the average LH value for HIV-negative individuals was 5.0 miu/mL (range: 4.22-7.24). LH value was significantly higher in HIV-positive individuals on ARVs when compared to HIV-negative individuals (p = 0.014). In Table 1, the average estradiol level for HIV-negative individuals was 38.20 pg/mL (range: 17.5-71.5 pg/mL), while the estradiol level for HIV-positive individuals not on ARVs was 52.54 pg/mL (range: 22.2-90 pg/mL) and the average estradiol levels for HIV-positive individuals on ARV was 36.0 pg/mL (range: 22.0-76.0 pg/mL). These results did not show significant differences across study groups. Table 2 revealed that the average antral follicular count was 10.57 in the HIV-negative participants, while the average antral follicular count in the HIV-positive group not on ARV was 10.10 and the antral follicular count in the HIV-positive group on ARV was 4.50. This value was significantly reduced in the study group who received ARVs when compared to the other study groups (p = 0.009).

**Table 1:** Comparing the average estradiol value in all three groups. The table shows that the average estradiol levels appear to be higher in the HIV-positive study group who are not receiving ARVs when compared to the average estradiol levels in the other study groups; however, this difference was not statistically significant on ANOVA (p = 0.56). ARV: antiretroviral drugs.

Group	N	Mean estradiol level (pg/mL)	Std. deviation	Confidence interval	ANOVA F-value	p-value
HIV-negative (group 3)	16	38.20	22.429	10.35 – 66.05	0.56	0.58
HIV-positive without ARV (group 2)	16	52.54	29.270	16.20 – 88.88		
HIV-positive with ARV (group 1)	16	36.00	27.674	1.64 – 70.36		

**Table 2:** Antral follicular count across the three cohorts. The table shows that the antral follicular count is significantly lower in the HIV-positive group receiving ARVs when compared to the HIV-positive group not on ARVs and the HIV-negative group using ANOVA. This difference is statistically significant (p = 0.009). ARV: antiretroviral drugs.

Group	N	Mean	Std. deviation	Confidence interval	ANOVA F-value	p-value
HIV-negative (group 3)	16	10.57	5.612	7.33 – 13.81	21.28	0.009
HIV-positive without ARV (group 2)	16	10.10	5.567	6.12 – 14.08		
HIV-positive with ARV (group 1)	16	4.50	3.631	2.19 – 6.81		

## Discussion

The study groups were well matched for age, thus minimizing any age-related bias in follicular counts since fertility and reproductive potential are known to decline with increasing age [15]. Of further note, the participants across the three study groups did not smoke or consume alcohol. Smoking and alcohol consumption would have been cofounders in this study, as binge drinking has been shown to be associated with premature ovarian failure and depletion of ovarian reserve [16]. Furthermore, female smoking has been shown to be associated with the depletion of ovarian reserve [17].

The participants in this study were immunologically and virologically stable, hence the influence of the pathology of the disease condition cannot be considered as a cofounder influencing the results. The average CD4 values across the study population who were HIV positive was 725 cells/mm3 and the average viral load was less than 20 copies per mL (undetectable viremia). These patients were therefore in a good state of health. The participants using efavirenz-based HAART in this study had used it for almost 3.5 years (39 months) years, reflecting long-term usage. This therefore is a significant difference between group 1 and the other two groups, when the results are interpreted. In these cohorts of participants, serum levels of FSH, LH, and estradiol were evaluated during the follicular phase. Serum levels of these hormones are known to fluctuate during the menstrual cycle, hence values used in the evaluation of patients are best measured during the follicular phase of the cycle when follicular recruitment has taken place [18]. The mean FSH value for the research participants who were HIV-positive on HAART (ARVs) was higher than the other two cohorts. This was statistically significantly raised compared to the control group 3 (p = 0.039), but not compared to the untreated HIV-positive group (p = 0.222).

A normal FSH value in reproductive-aged women during the follicular phase is 3.77.9 mIU/mL [19]. The FSH value is a predictor of ovarian reserve and should directly correlate with the values of antral follicular count (AFC). The average FSH value in patients who were HIV positive and were taking HAART (ARVs) was higher than the normal range. The patients who were HIV positive and on HAART (ARVs) had abnormal FSH values when compared to the group who were HIV positive and not on medications and the HIV-negative group. While all our patients were well within the reproductive age range, it should be noted that FSH values above 30.6 mIU/mL are considered menopausal [19] and that FSH values are a late predictor of ovarian reserve, while anti-Mullerian hormone (AMH) levels and AFC are early and more accurate predictors of ovarian reserve [20]. Santoro and co-workers studied the factors affecting reproductive hormones in HIV-positive women with substance abuse who were within the reproductive age range. The authors concluded that the use of ARVs in this cohort of patients was associated with higher FSH, LH, and estradiol when compared with controls [21].

Estimation of serum levels of estradiol for reproductive function is usually measured during the follicular phase of the cycle when there is follicular recruitment. The normal range for estradiol in human females is 27-123 pg/mL [19]. In this study, the average serum level of estradiol in HIV-positive participants receiving ARVs was lower than the average serum level of estradiol in the other two cohorts. The average serum level of estradiol was 36.00 pg/mL for HIV-positive patients receiving ARVs, while that of HIV-positive patients not on ARVs was 52.50 pg/mL. The average serum level of estradiol for respondents who are HIV-negative was 38.20 ng/mL. These differences were, however, not statistically significant. A low level of estradiol is present in premature ovarian failure and menopause. Estradiol levels tend to exhibit an inverse relationship with the levels of FSH and a direct relationship with the levels of AMH [22]. This pattern is not always the case as serum levels of both estradiol and FSH are late and less than accurate predictors of ovarian reserve [23]. Estradiol levels below 40 pg/mL are reflective of a value close to that in the menopausal range.

The normal range of LH during the follicular phase is 1-18 miu/mL [24]. LH is not a reliable predictor of ovarian reserve and reproductive potential because of its fluctuation over the menstrual cycle duration and because of the broad range of normal values. LH is, however, useful when interpreted in conjunction with FSH when making a diagnosis of polycystic ovarian syndrome (PCOS). In this regard, the LH/FSH ratio becomes relevant. The normal value is 1:1, but in patients with PCOS, the ratio becomes 2:1 or 3:1 due to the elevated values of LH [18]. We found that the average LH value was more elevated in the HIV-positive group than in the HIV-negative group. The elevation was more marked in the HIV-positive group on ARVs than in the HIV-positive group not on ARVs. This difference was statistically significant (p = 0.014). Elevated LH levels when used in conjunction with FSH levels suggest anovulation [21].

In this study, while the level of AMH was not estimated, the AFC test was performed as AFC is another accurate predictor of ovarian reserve. AFC is a marker for ovarian reserve and a reflection of the fertility potential of human females. It is the gold standard for the measurement of the ovarian reserve of human females [25]. It can be used in isolation in the determination of patients who will require egg donation in preparation for in vitro fertilization (IVF) using a donor egg [25].

AFC measurement in this study was done with the aid of a four-dimensional color Doppler ultrasound machine with a transvaginal transducer. Color Doppler ultrasound is superior to plain ultrasound machines because it can discriminate follicles from blood vessels. The study revealed that AFC was significantly lower in HIV-positive individuals receiving ARVs when compared to the participants in the other cohorts (p = 0.009). AFC values have been shown to be a better predictor of hyperstimulation in IVF cycles when compared to AMH [26,27].

The reduction in AFC in the human population was also reflected in the findings obtained from the estimation of the FSH levels of study participants, as AFC is a marker of ovarian reserve and correlates with FSH [28].

AFC estimation is undertaken during the follicular phase of the menstrual cycle, usually between days seven and 10, as this is the period of the cycle where follicular recruitment occurs. Values above eight follicles per ovary are considered normal. The normal range is between eight and 16 antral follicles. Of note, patients with PCOS are known to have high AFC [29]. AFC is usually over 18 follicles per ovary in patients with PCOS.

In the past, it was thought that HIV could negatively impact the values of AFC [30] but it was not clear if this was because of the impact of the disease or other confounding factors. Our earlier works demonstrated impaired folliculogenesis in a rodent model of HAART (ARVs), using an efavirenz-based regimen, and demonstrated a higher occurrence of menstrual irregularities in those rodents receiving this regimen [12,13]. This current work appears to point to a similar trajectory as reduced AFC and elevated FSH levels in HIV-positive women receiving efavirenz-based HAART are suggestive of reduced ovarian reserve in these women. This is further corroborated by the fact that age-matched controls of women who are HIV positive and not receiving HAART and HIV-negative women did not show similar findings. When all these findings are put together, this work has demonstrated compromised folliculogenesis and dysfunctional reproductive hormone profile in HIV-positive women within the reproductive age receiving efavirenz-based ARVs.

The limitation of this study is that the cross-sectional nature of the study did not allow for repeated evaluation of the reproductive hormone profile and AFC of the participants of the study. Repeated evaluation of these reproductive parameters would have better characterized the changes in the reproductive potential of the participants in the study over a defined period. Furthermore, other limitations include the shorter time frame of the study, the older age of the participants, and the influence of the BMI of the participants. Evaluation of reproductive parameters in women using ARVs over a prolonged period will more accurately define patterns and associated factors influencing the reproductive parameters.

## Conclusions

The findings from this study have shown that prolonged use of ARVs is possibly associated with diminished ovarian reserve as demonstrated in the compromised follicular development and dysfunctional reproductive hormone profile seen in HIV-positive women within the reproductive age who are receiving ARVs. The fact that age-matched controls who are HIV-positive and not yet on ARVs and HIV-negative women did not show similar profiles is an indication that these medications may be responsible for the abnormal reproductive profile in these groups of women. Caregivers should consider a review of the current regimen in use for the management of women within the reproductive age range who are being managed with efavirenz-based combinations with a view to transitioning these patients to ARVs with less deleterious effects to the reproductive potential of these group of women.
